# Impacts of Thermal Atomic Layer-Deposited AlN Passivation Layer on GaN-on-Si High Electron Mobility Transistors

**DOI:** 10.1186/s11671-016-1335-7

**Published:** 2016-03-10

**Authors:** Sheng-Xun Zhao, Xiao-Yong Liu, Lin-Qing Zhang, Hong-Fan Huang, Jin-Shan Shi, Peng-Fei Wang

**Affiliations:** State Key Laboratory of ASIC and System, School of Microelectronics, Fudan University, 220 Han Dan Road, Shanghai, China

**Keywords:** AlGaN/GaN HEMT, 2-DEG, ALD, Passivation

## Abstract

Thermal atomic layer deposition (ALD)-grown AlN passivation layer is applied on AlGaN/GaN-on-Si HEMT, and the impacts on drive current and leakage current are investigated. The thermal ALD-grown 30-nm amorphous AlN results in a suppressed off-state leakage; however, its drive current is unchanged. It was also observed by nano-beam diffraction method that thermal ALD-amorphous AlN layer barely enhanced the polarization. On the other hand, the plasma-enhanced chemical vapor deposition (PECVD)-deposited SiN layer enhanced the polarization and resulted in an improved drive current. The capacitance-voltage (C-V) measurement also indicates that thermal ALD passivation results in a better interface quality compared with the SiN passivation.

## Background

AlGaN/GaN high electron mobility transistor (HEMT) is promising for high frequency, high power density, and high temperature applications owing to its superior material properties such as wide bandgap (3.4 eV), high breakdown field (2 × 10^6^ V/cm), high thermal stability, and its 2-D electron gas (2DEG) channel [1, 2]. In order to realize GaN HEMTs on the mainstream 8-in. wafer, GaN-on-Si HEMTs have been intensively investigated. In the typical structure of GaN-on-Si HEMTs, the crystalline defect density is high because of the lattice mismatch of two material systems. The electron trapped at the AlGaN surface can cause surface leakage current and drain current collapse effects [2–4]. Therefore, passivation techniques have been widely applied for filling these traps [5–8].

ALD-grown AlN is a wide band-gap material recently reported as a new choice to passivate AlGaN/GaN HEMTs for its good isolation ability and high quality interface with AlGaN/GaN [3, 9]. However, most of the studies of the AlN passivation layer were grown by plasma-enhanced ALD (PEALD). In this work, we develop the thermal ALD technique and use it as a passivation layer. The plasma-free process leads to a simpler growth process and prevent possibly plasma-induced damage. A relatively larger film thickness is also applied to keep the traps on the new surface away from the 2DEG channel. For comparison, plasma-enhanced chemical vapor deposition (PECVD)-deposited SiN passivation layer is also grown on AlGaN/GaN HEMTs. The output characteristics of devices using the two different passivation techniques are compared and discussed in this work.

## Methods

AlGaN/GaN HEMTs were fabricated on samples on Si(111) substrate with GaN buffer layer. A 25-nm Al_0.25_Ga_0.75_N layer barrier and a 3-nm undoped GaN cap layer were used. First, mesas were defined by BCl_3_/Ar-based reactive ion etching (RIE). After that, Ti/Al/Ni/Au (20/120/60/50 nm) stack were deposited by electron beam evaporation (EBE) followed by a rapid thermal anneal at 840 °C for 30 s to form the ohmic contact. Then, Schottky metal gates of Ni/Au (20/200 nm) were also deposited by EBE. The gate length, gate-to-drain spacing, gate-to-source spacing of fabricated HEMTs are all 1.5 μm. The output and transfer characteristics of the basic HEMT without passivation were then measured.

**Fig. 1:**
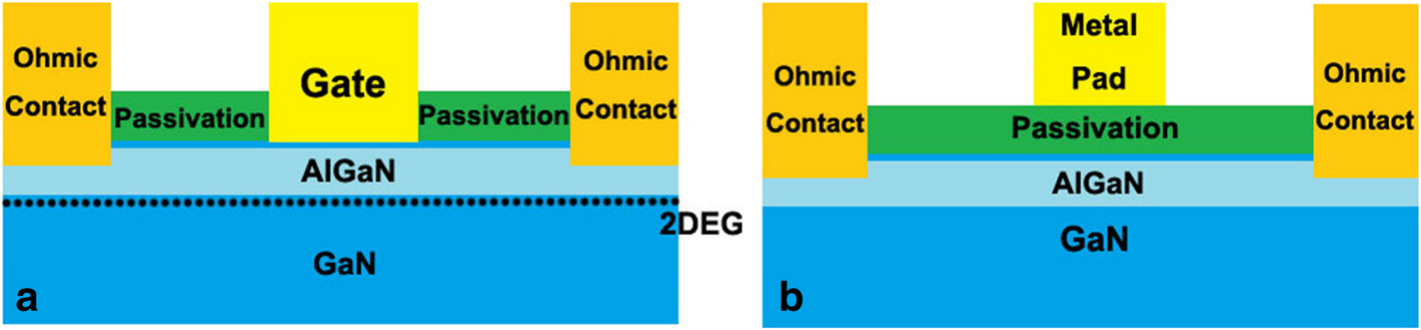
Schematic diagram of the structure of AlGaN/GaN HEMTs (**a**), and C-V test structure (**b**)

Finally, the passivation layer was deposited, and the contact windows were opened. The 30-nm AlN passivation layer was grown by BENEQ TFS200 ALD system with NH_3_ and trimethylaluminum precursors. During the reaction, the chamber is maintained at 400 °C. After the deposition of passivation layer, the contact windows were opened by RIE with BCl_3_/Ar. The device structure with passivation is shown in Fig. 1a. For comparison, the 110-nm SiN passivation layer was deposited by PECVD with N_2_/SiH_4_/NH_3_ at 350 °C. Then, the contact windows were opened by RIE with CF_4_. After that, the metal pads of Ni/Au (20/200 nm) for capacitance-voltage (C-V) measurements were deposited by EBE. The electrical characteristics were then collected and compared with the data before passivation. The capacitor structure using the passivation layer as dielectric layer is shown in Fig. 1b.

## Results and Discussion

**Fig. 2:**
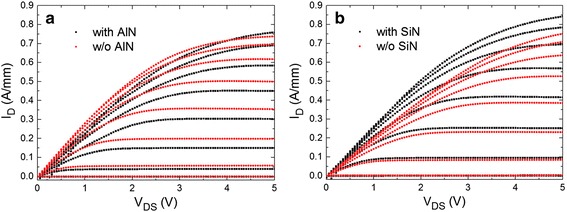
Comparisons of I_D_-V_DS_ results at V_GS_ varied from −5 to 3 V (step = 1 V) of the devices before and after **a** thermal ALD-grown AlN, **b** PECVD-deposited SiN passivation

Figure 2 shows the output characteristics of AlGaN/GaN HEMTs with/without passivation layer. The drive current of SiN-passivated device increases obviously from 0.751 to 0.843 A/mm (V_GS_ = 3 V, V_DS_ = 5 V) while that of AlN-passivated device increases slightly from 0.738 to 0.759 A/mm (V_GS_ = 3 V, V_DS_ = 5 V).

**Fig. 3:**
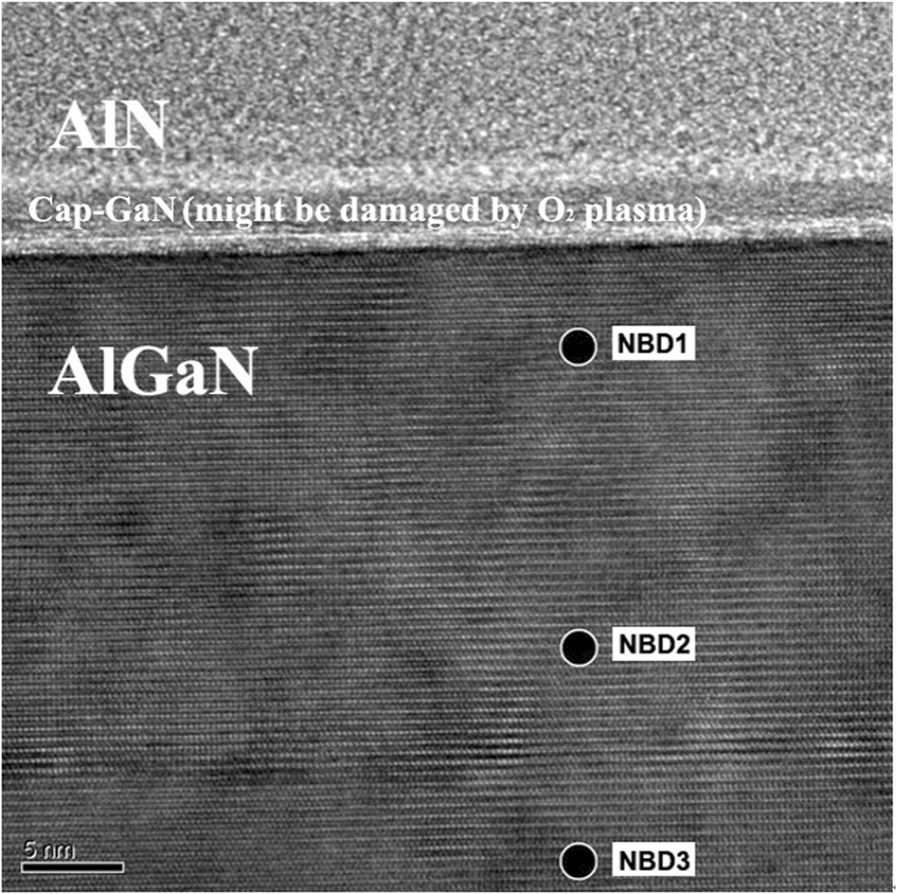
NBD sampling positions of AlN-passivated sample

**Fig. 4:**
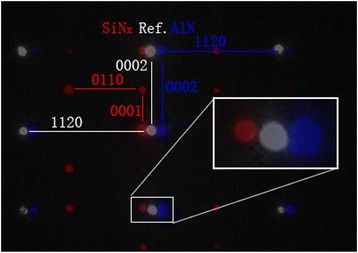
Overlapped NBD results of SiN-, AlN-passivated, and unpassivated sample at position NBD2

In order to explain the different behavior of devices using AlN and SiN passivation, nano-beam diffraction (NBD) was applied on the passivated device to detect the strain-caused deformation of AlGaN layer [10]. An unpassivated sample is used as the reference. The sampling positions of the AlN-passivated sample are shown in Fig. 3. Similar positions are chosen for the SiN-passivated samples. Figure 4 shows the overlapped NBD results at position “NBD2” indicated in Fig. 4. The compressed *c* axis of AlGaN in the SiN-passivated sample can be observed. While, the NBD results of AlN-passivated samples have no clear difference. The specific deformation results of all three positions in the two samples are compared with the reference and shown in Table 1. It can be seen that a tensile strain was applied on the AlGaN layer for the SiN-passivated sample, while almost no strain was detected applied on the AlGaN layer for the AlN-passivated samples.

**Table 1:** Varieties of *c* axis of AlGaN/GaN layer after passivation

Position	SiN passivated (%)	AlN passivated (%)
NBD1	−0.33	≈0
NBD2	−0.33	≈0
NBD3	−0.20	≈0

**Fig. 5:**
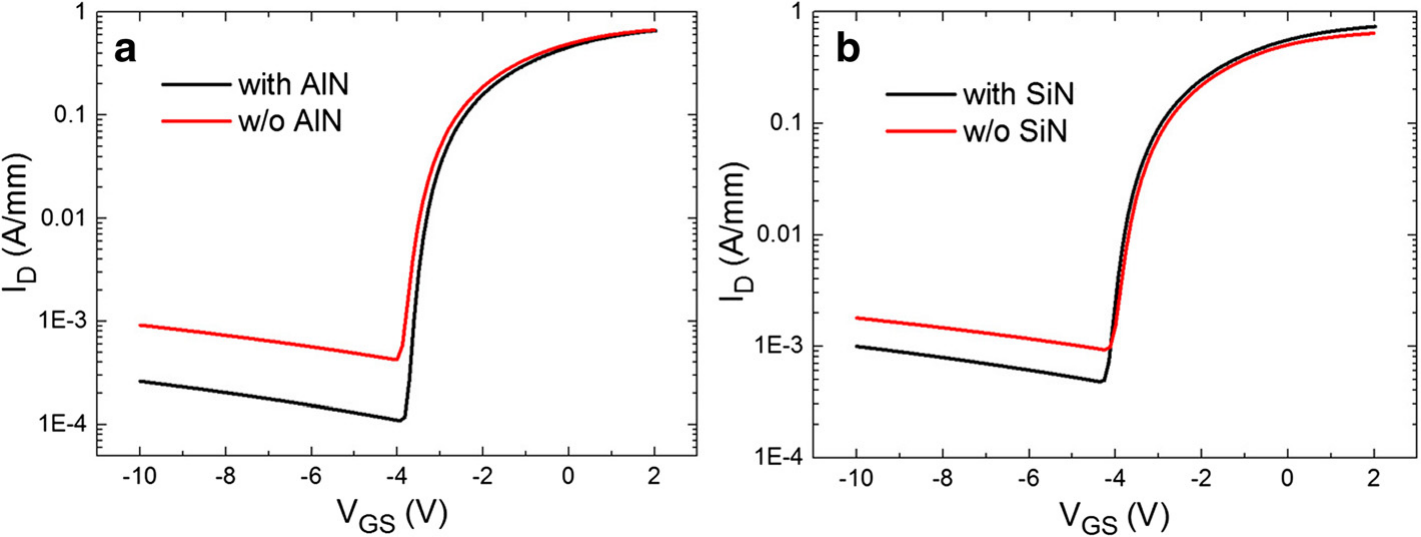
Comparisons of I_D_-V_GS_ results at V_DS_ = 4 V of the devices before and after **a** thermal ALD-grown AlN, **b** PECVD-deposited SiN passivation

Figure 5 shows the comparison of the transfer characteristic before/after AlN and SiN passivation. It can be seen that both two passivation techniques can suppress the off-state leakage. Wherein, the off-state current of AlN-passivated device dropped more significantly from 4.98 × 10^−4^ to 1.29 × 10^−4^ A/mm (V_GS_ = −5 V, V_DS_ = 4 V) than the SiN passivated one from 10.2 × 10^−4^ to 5.20 × 10^−4^ A/mm (V_GS_ = −5 V, V_DS_ = 4 V).

In Fig. 4, the drive current of AlN-passivated device is almost unchanged compared with the sample before AlN passivation. That means the increase of sheet concentration caused by the polarization-induced positive charge at the AlN/capGaN interface was not obvious [11]. As indicated in Table 1, the strain caused by the AlN passivation layer is negligible. In the previous work of Huang et al., crystalline AlN layer was used [9]. The band gap and charge concentration of amorphous AlN are both lower than the crystalline AlN [12, 13], so that there are lower polarization and fewer charges at the interface. However, the lower polarization also means higher barrier and lower leakage current at the AlN/cap-GaN interface. The leakage current of amorphous AlN layer is lower than crystalline AlN layer as well [14].

On the other hand, an enhancement of the drive current in the SiN-passivated devices is observed. Depending on the deposition conditions, the SiN deposition process can apply tensile or stress strain [15]. As shown in Table 1, the NBD analyses results indicate that a tensile strain was applied on the AlGaN layer. Therefore, piezoelectric polarization is enhanced, and the carrier density of 2DEG is increased [9, 16]_._

**Fig. 6:**
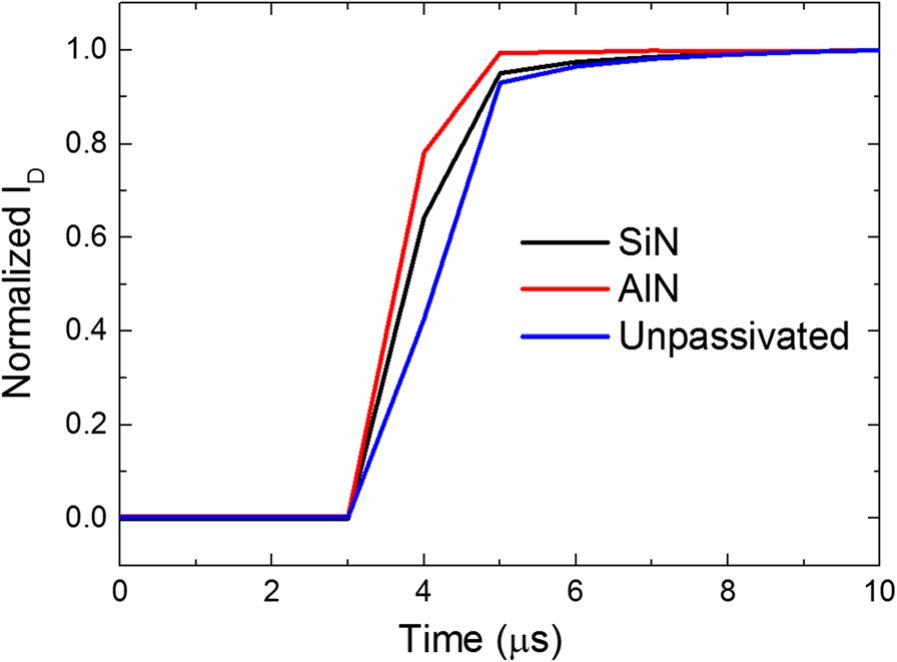
Normalized response I_D_ when V_GS_ pulsed from −6 to −2 V at V_DS_ = 5 V of SiN-passivated sample, AlN-passivated sample, and unpassivated sample

The ability of quick switch of the SiN-passivated sample, AlN-passivated sample, and unpassivated sample were tested. The V_GS_ was pulsed from −6 to −2 V at V_DS_ = 5 V, then the normalized response *I*_D_ of the three samples were collected and shown in Fig. 6. The AlN-passivated sample performed best in quick switch, which indicated that it contains the fewest trapped charges to obstruct the gate control among these three samples.

Capacitance-voltage (C-V) test was applied on the two types of passivation layers to detect the difference in filling interface traps and the qualities of passivation layers to describe this difference [17]. 30-nm thermal ALD-grown AlN and 30-nm PECVD-deposited SiN were tested.

**Fig. 7:**
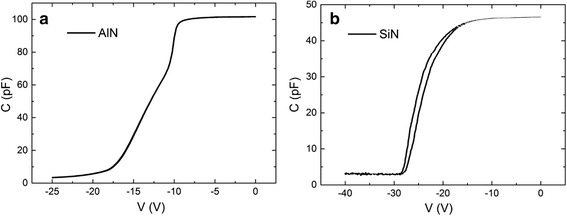
Results cycled C-V test at 1 MHz of **a** thermal ALD-grown AlN, **b** PECVD-deposited SiN-passivated samples

Figure 7 shows the results of C-V test at 1 MHz frequency. The capacitor structure is shown in Fig. 1b. In Fig. 7b, a hysteretic effect was seen between the low-to-high and high-to-low voltage scan results of the SiN-passivated device. The hysteretic effect of C-V scanning results was caused by the unsealed interface traps and defects inside the passivation. In Fig. 7a, the C-V scan results of AlN-passivated device has no hysteretic effect. That means most of the interface traps were sealed by the AlN passivation layer.

## Conclusions

The thermal ALD-grown amorphous AlN passivation layer owns a satisfactory interface state and film quality. This passivation technique performs better in sealing interface traps and reducing the off-state leakage than the PECVD-deposited SiN. Low polarization is observed for the thermal ALD-grown amorphous AlN passivation layer. Amorphous AlN is also promising as gate dielectric for AlGaN/GaN HEMT because of its predictable lower leakage than the crystalline one.
